# Modeling Presenilin-Dependent Familial Alzheimer's Disease: Emphasis on Presenilin Substrate-Mediated Signaling and Synaptic Function

**DOI:** 10.4061/2010/825918

**Published:** 2010-07-20

**Authors:** Angèle T. Parent, Gopal Thinakaran

**Affiliations:** ^1^Department of Neurobiology, The University of Chicago, 924 East 57th Street, Chicago, IL 60637, USA; ^2^Department of Neurology, The University of Chicago, 924 East 57th Street, Chicago, IL 60637, USA

## Abstract

Mutations in *PSEN* genes, which encode presenilin proteins, cause familial early-onset Alzheimer's disease (AD). Transgenic mouse models based on coexpression of familial AD-associated presenilin and amyloid precursor protein variants successfully mimic characteristic pathological features of AD, including plaque formation, synaptic dysfunction, and loss of memory. Presenilins function as the catalytic subunit of *γ*-secretase, the enzyme that catalyzes intramembraneous proteolysis of amyloid precursor protein to release *β*-amyloid peptides. Familial AD-associated mutations in presenilins alter the site of *γ*-secretase cleavage in a manner that increases the generation of longer and highly fibrillogenic *β*-amyloid peptides. In addition to amyloid precursor protein, *γ*-secretase catalyzes intramembrane proteolysis of many other substrates known to be important for synaptic function. This paper focuses on how various animal models have enabled us to elucidate the physiological importance of diverse *γ*-secretase substrates, including amyloid precursor protein and discusses their roles in the context of cellular signaling and synaptic function.

## 1. Introduction

Mutations in *PSEN1* and *PSEN2 *genes, which encode polytopic proteins termed presenilin 1 (PS1) and presenilin 2 (PS2), respectively, cause autosomal dominant early-onset familial Alzheimer's disease (FAD) [1]. Both PS1 and PS2 proteins (PS) share about 63% homology with the highest similarity in the transmembrane domains where most of the FAD-linked mutations are found [2, 3]. Since the first report of mutation in the *PSEN1* on chromosome 14, about 170 mutations have been identified, making mutations in *PSEN1* the most common cause of autosomal dominant early-onset AD [4]. In the case of *PSEN2*, 18 mutations have been reported so far, although not all have been confirmed to be pathogenic [2, 5]. As a probable explanation for the disparity between the two genes, defects in *PSEN2* function may be offset by the normal function of its homolog *PSEN1*. In support of this view, *PSEN2* null mice do not exhibit the phenotypic and functional defects seen in mice lacking *PSEN1 *gene. *PSEN1 *knockout (KO) mice are lethal, and disruption of *PSEN2* and *PSEN1* genes causes earlier embryonic lethality compared to *PSEN1* KO [6–10]. As supported by mouse model studies, it appears that PS1 contributes largely to total *β*-amyloid (A*β*) production in the brain [11, 12]. 

PS is the catalytic subunit of *γ*-secretase, the enzyme responsible for intramembraneous cleavage of amyloid precursor protein (APP) to generate peptides. FAD-linked PS variants enhance the production of highly fibrillogenic A*β*42 peptides that are deposited early in the brains of patients with AD [13]. PS is ubiquitously expressed in the nervous system and peripheral tissue and found localized in secretory and endocytic organelles in all cell types, as well as synaptic structures in neurons [14, 15]. As predicted from its broad pattern of expression, PS's function extends far beyond processing of APP and the pathogenesis of AD. For example, PS's catalytic function is required for intramembraneous *γ*-secretase cleavage of Notch receptors, which releases the Notch intracellular domain (NICD). Nuclear signaling mediated by NICD is essential during mammalian development; mice with ablated *PSEN1* alleles die in late embryogenesis and exhibit phenotypes reminiscent of mice lacking *Notch 1 * [6, 7]. Thus, PS-dependent activation of Notch signaling is essential for early development. Transgenic expression of FAD-linked mutant PS1 fully rescues the developmental phenotypes in mice with *PSEN1 *deficiency [16, 17], supporting the notion that FAD-linked PS1 variants are functional, but acquired deleterious properties that have profound pathophysiological consequences. Candidate approaches and proteomic studies have identified a wide spectrum of type I membrane proteins that undergo *γ*-secretase cleavage, including Notch ligands, Deleted in Colorectal Cancer (DCC), and cadherins (reviewed in [13, 18–21]). Uniformly these substrates all undergo an ectodomain shedding by *α*-secretases, which in many cases is triggered by the binding of extracellular ligands. Interestingly, several noncatalytic *γ*-secretase-independent functions have been assigned to PS, such as its role in regulating intracellular calcium homeostasis (reviewed in [22]).

Synapses are continuously reconfigured, both structurally and functionally, during embryonic development and throughout adult life, forming the basis for learning and memory [23, 24]. Neuronal inability to exhibit such plastic changes has been proposed to be a root cause for various psychiatric and neurodegenerative disorders such as AD [23, 25, 26]. Not surprisingly, the duration and severity of cognitive impairments in AD patients closely parallels the extent of synaptic loss, leading to the notion that synaptic dysfunction is a critical element in the pathophysiology of AD [27]. Notably, memory and cognitive decline observed in AD patients correlate better with the synaptic pathology than either A*β* plaque load or tangle density, and synapse loss appears to precede neuronal degeneration. Details on how synaptic organization is altered in AD patients are beginning to emerge. Findings from several laboratories suggesting that A*β* might play a critical role in synaptic dysfunction have added significant information to the traditional amyloid cascade hypothesis of AD [28, 29]. A*β* can affect synaptic transmission [30–33], synaptic protein localization [34], AMPA and NMDA receptor trafficking [35, 36], and spine formation [35, 37–39].

FAD-linked mutations in PS1 were originally thought to enhance the production of A*β*42 peptides by a gain-of-function mechanism. However, it is becoming clear that FAD-linked PS1 variants also exhibit partial-loss-of-enzymatic-function observed as diminution of A*β*40 peptide production and defects in the extent of processing certain other transmembrane substrates (reviewed in [40, 41]). For example, FAD-linked PS1 mutations are thought to attenuate *γ*-secretase processing and generate reduced levels of the intracellular domains of APP, Notch, N-cadherin, EphB2, and EphA4 [42–45]. Taken together, it is plausible that FAD-linked mutations in PS1 exert pathophysiological effects on the synapses by elevating A*β*42 levels and by A*β*-independent mechanisms involving altered processing of *γ*-secretase substrates involved in synaptic function. This paper discusses findings from various animal models that reveal the role of PS and FAD-linked PS mutations in synapse formation and function.

## 2. PS Animal Models

Several mouse models (reviewed in [46]; see http://www.alzforum.org/res/com/tra/) and a few rat models [47–50] have been developed in order to recapitulate the main pathological features of AD and elucidate the mechanisms by which FAD-linked PS mutations contribute to AD pathogenesis. A variety of mouse models have been characterized such as mice expressing FAD-linked PS variants harboring point mutations or deletion mutation [51, 52], and FAD-linked *PSEN1* knock-in (KI) [M146V variant [53], I213T variant [54] and P264L variant [55]], and ΔE10 loop deletion KI [56]. These FAD-PS1 single transgenic or KI mouse models do not exhibit significant A*β* deposition in the brain. Therefore, the phenotypes described in these FAD-PS1 single transgenic mice are not due to classical A*β* pathology. In an attempt to reproduce more closely the human AD pathology, *PSEN1* KI coexpressing APP “Swedish” mutant and hyperphosphorylated tau mutants have been made [57].

In order to study the physiological function of PS, KO models of *PSEN1* and *PSEN2* [6–10], *PSEN1* conditional KO [58–60], as well as double *PSEN1* and *PSEN2* conditional KO [61] mice have also been created. In order to examine amyloid pathology, transgenic mice expressing APP mutants in a PS null background have been developed; such as *PSEN1* conditional KO coexpressing APP V717I variant [60] and APP V717F variant [62]. In these models, A*β* deposition is attenuated by the lack of PS1 expression and consequent loss of *γ*-secretase activity. 

Besides their utility in examining proteolytic processing of APP into A*β*40 and A*β*42 peptides *in vivo* and phenocopying pathological hallmarks of AD (amyloid deposition and tau phosphorylation), these models have been extensively used to examine changes in synaptic transmission, synaptic plasticity, and associated signaling. In addition, several groups have generated *Drosophila* models (reviewed in [63]), and *Caenorhabditis elegans* models (reviewed in [64]) expressing human PS1 or PS2 bearing FAD-linked mutations, in an effort to understand mechanistic contribution of PS to AD pathology and neuronal dysfunction.

## 3. PS and Cellular Substrates of Memory

Synaptic transmission and long-term potentiation (LTP) contribute to several forms of memory storage. Using slice preparations from transgenic mice, we and others have demonstrated that expression of FAD-linked PS1 does not alter basal synaptic transmission, but leads to higher degree of LTP induction in the hippocampus ( [57, 65–69] reviewed in [14]). However, one group has reported impairment of synaptic transmission associated with an increase of paired-pulse facilitation, an index of presynaptic release, in neurons of 6 month-old *PSEN1* M146V KI mice [57]. LTP induction by high-frequency stimulation in hippocampal CA1 area was also enlarged in this animal model [57]. Interestingly, in *PSEN1* M146V KI animal model, LTP induced by carbachol (a muscarinic agonist) was reduced in CA1 hippocampal area, suggesting that FAD-linked PS1 variant might interfere with cholinergic cellular cascades as well [70]. The KI mouse models allow us to examine the functional properties of molecules associated with pathology when they are expressed at endogenous levels without any alteration in their spatial or temporal pattern of expression. Therefore, KI animal models give us the opportunity to rule out pathophysiological consequences (such as protein misfolding) associated with aberrant overexpression of proteins associated with human genetic disorders. 

Interestingly, it has been described that the lack of PS function or overexpression of PS1 mutant was also associated with changes in presynaptic function. We have observed an increase of spontaneous miniature excitatory postsynaptic current in cortical neurons isolated from *PSEN1* KO mice [71], while others have reported that expression of mutant PS1 in cultured hippocampal neurons depresses synaptic transmission by reducing the number of synapses [72]. Another group has also observed that PS1 deficiency increases synaptic release and affects the number and docking of synaptic vesicles [69]. It was also shown that basal transmitter release was increased at the neuro-muscular junction in Drosophila lacking PS expression [73]. However, even though basal synaptic transmission seems to be intensified in this later model, synaptic strength and plasticity were impaired after posttetanic potentiation [73]. As a likely consequence, associative learning ability was also impaired. In parallel, it has been reported that LTP induction declines more rapidly in CA1 hippocampal area of mice with only one allele of *PSEN1* [74]. In agreement with these observations, it has been recently found that a CA3-dependent presynaptic form of LTP in the hippocampus was attenuated in double *PSEN1* and *PSEN2* conditional KO mice [75]. Intriguingly, single *PSEN1* conditional KO mice do not show major changes in brain plasticity, suggesting that expression of PS2 might be sufficient to overcome the 60–80% loss of PS1 in the forebrain of these animals [59]. 

What can we learn from these animal models? First of all, it becomes apparent from these studies that PS is an essential element for the normal synapse function. Second, it becomes evident that PS dosage is a critical component for PS-dependent cellular function(s). Indeed, PS1 expression is developmentally regulated in rodent brain, reaching a peak of expression during the critical period of synaptogenesis between postnatal days 7 to 14 [76]. Accordingly, we can stipulate that PS-dependent substrates expressed during embryogenesis or early in development may significantly contribute to synaptic physiology. In this regard, it also remains to be established whether differences in PS-dependent proteolysis of developmentally regulated molecules might underlie changes in synaptic function later on in life. A well-known example is a condition where stress-induced early life biochemical events influence life-span changes in cognitive function and AD-associated abnormalities [77]. Accordingly, it has been proposed that age-related decline in cortical cholinergic function in AD patients might have developmental origins [78]. Finally, it has also been speculated that PS-dependent modulation of signaling pathways that are important in development may contribute to the neurodegenerative process [79]. Taken together, studies from various laboratories suggest that PS is specifically involved in cellular component(s) necessary for synaptic transmission and plasticity, and that FAD-linked mutations in PS1 may disrupt the normal cascade of synaptic events.

## 4. PS and Synapse Formation

A distinct feature of the nervous system is the intricate network of synaptic connections among neurons. The changes in the strength and efficacy of existing synapses, as well as remodeling of connectivity through the loss and gain of synapses in the neuronal network, are believed to be the basis of learning and memory in the brain. Interestingly, LTP has been associated with the increase in spine formation and spine head growth, whereas long-term depression (LTD) has been associated with spine shrinkage and retraction [80]. The morphology of dendritic spines is known to change in response to several factors including learning, age, hormones, and disease conditions [81]. In addition to their morphological plasticity, spine-like protrusions also display rapid motility, changing shape and size in a matter of seconds to minutes. This morphological plasticity suggests that long-term memory might be encoded by alterations in spiny structures and associated synaptic contacts [82]. Collectively, these events are critically important in synaptogenesis, in modulating of existing synapses, as well as in long-term synaptic plasticity [83, 84]. It has been reported that A*β* is closely associated with a decrease of spine formation and motility [35, 37, 85]. Overproduction of A*β* in PS mutant transgenic mice coexpressing the “Swedish” APP mutant causes age-associated decrease of synaptic excitability [57, 86, 87] and spine collapse [38, 88]. However, it has also been reported that acute A*β* application (less than 4 h) was associated with an increase of filopodia and growth cones in hippocampal cultures [89]. In support of this idea, it was shown that application of low levels of A*β* is associated with an increase of LTP, whereas higher concentration of A*β* reduced synaptic potentiation [32, 90]. Collectively, these observations suggest that A*β* might have dual roles on synapse formation. Conflicting results have also been observed in regard to spine morphology in neurons lacking PS expression. Treatment with Compound E, a *γ*-secretase inhibitor (10 nM; 24 h), produced an increase of spine-like protrusions in isolated neurons [71, 91]; whereas the density of spines was found to be decreased upon prolonged treatment with the same inhibitor (50 nM; seven days) [45]. In addition, neurons lacking both PS1 and PS2 expression have marked diminution in spine density [45]. To further support the effect of *γ*-secretase inhibition on dendritic spines, recent *in vivo* study showed that *γ*-secretase inhibitor treatment in wild-type mice significantly reduced the number of spine density in somatosensory cortex, while *γ*-secretase inhibitor treated APP null mice did not exhibit any effect [92]. These findings suggest that APP-dependent mechanism may underlie the PS-dependent morphological changes observed. The apparent discrepancy between inhibitor treatment and loss of PS expression on spine density may be also due to differential effects of inhibitors that target mainly *γ*-secretase and genetic inactivation of PS that results in reduced *γ*-secretase-dependent and -independent function. All together, these observations support the idea that PS gene dosage and the level of expression may differentially influence synaptic morphology.

Although the molecular mechanisms that underlie these morphological changes are not completely understood, emerging evidence supports at least two important signaling pathways that have been linked to dendrite spine formation and AD etiology: (1) cAMP-dependent activation of PKA has been shown to be critical for the maintenance of the late phase of LTP, and downstream phosphorylation of CREB has been linked to formation of new spines [93]. Interestingly, it has been shown that A*β* inhibits PKA/CREB pathway [94], (2) the Rho family of small GTPases, well-known regulators of the actin cytoskeleton, has profound influence on spine formation. Among the members of this family Rac1, Cdc42, Rnd1, and Ras promote spine formation and growth, whereas Rap and RhoA induce shrinkage and loss of spines [80, 95]. p21-activated kinase (PAK) is a downstream signaling effector of the Rho/Rac family of small GTPases and has been shown to be associated to spine formation and memory consolidation [96]. A role of PAK in cognitive deficits of AD has also been reported [97]. 

A recent paper by Shuai and colleagues [98] suggests that the act of forgetting might also be linked to activation of the Rac pathway, using a simplistic model of olfactory learning in the fruit fly *Drosophila*. With the help of genetic manipulation, they were able to distinguish changes in Rac activity during passive memory decay, interference learning, and reversal learning, which are three different forms of forgetting events. In *Drosophila* olfactory memory model, it appears that cAMP/PKA and Rac/PAK-dependent memory acquisition and forgetting events are independent, as suggested by this group and others [98, 99]. In a more complex system, as it has been proposed in the mammals, it seems that memory consolidation might mechanistically require both pathways [96, 100, 101]. As demonstrated by several groups, Rac signaling cascade in the brain is directly linked to an increase of spine formation through subsequent activation of PAK leading to F-actin polymerization and changes in membrane morphology. Besides the known involvement of cAMP/PKA/CREB activation cascade, Rac/PAK-dependent cellular events also appear to be intimately associated with the process of memory consolidation, at least in rodents.

It is very exciting to think that perhaps similar cellular pathways as the one described above may be relevant to human disorders associated with memory dysfunction. One of the known hallmarks of AD is that patients do forget recent events, therefore, they are unable to consolidate their new memory. In our lab, we have shown that the lack of PS function or expression in cortical neurons produced an increase of steady-state levels of CREB and Rac/PAK cascade activation, which was also associated with an increase of spine-like protrusions [91]. Even though our study shows increase of phosphorylated CREB especially in dendritic area, transcriptional CREB activity was not directly determined in this experiment. More recently, Shen and collaborators have shown that CREB transcription was indeed reduced in PS deficient neuron through PS-independent mechanism [102]. Are these signaling events meaningful in the context of AD? Perhaps. As discussed above, recent studies support the idea that FAD-linked mutations in PS1 might cause a partial loss of function [40, 41]. It still remains to be determined whether Rac/PAK signaling is altered in neurons expressing FAD-linked PS1 variants. If this is the case, one might want to consider the possibility that changes in cAMP/PKA/CREB or Rac/PAK signaling in neurons might represent some of the earliest cellular dysfunctions that are relevant to synapse elimination and associated cognitive decline in AD.

## 5. PS-Dependent Substrate Signaling

*γ*-secretase-dependent PS function mediates transmembrane proteolysis of several substrates including APP, N- and E-cadherins, *γ*-protocadherin, CD44, DCC, ephrin/Eph receptors, leukocyte-common antigen related, nectin-1*α*, and syndecan (reviewed in [18, 20, 21]). Many of these substrates function as cell-adhesion molecules or cell surface receptors and are known for their diverse functions during development and are involved in axon guidance, neuronal outgrowth and synaptogenesis [103–113]. In addition, these molecules are also well known to be coupled to diverse intracellular signaling pathways [20, 44, 45, 108–110, 114–118]. It has been proposed that APP can affect synaptic function by its dual roles via its cell adhesive properties or through its putative receptor-like intracellular signaling components [112, 116, 117]. Indeed, it has been shown that accumulation of the APP intracellular domain can mediate a phosphoinositide-dependant calcium signaling [119]. Several other substrates of *γ*-secretase are also coupled with intracellular signaling events that can potentially influence synaptic function. For example, Eph receptors and N-cadherin are known to be coupled to Rac and CREB signaling, respectively [45, 115, 117, 120, 121]. Lack of EphB expression or kinase-defective EphB is associated with a reduction in glutamatergic synapses and abnormal spine development [120–122]. 

It has also been shown that three substrates of PS, namely ErbB4, *γ*-protocadherin, and leukocyte-common antigen related, are associated with PSD-95 clustering at the synapse [123, 124] and AMPA receptor function [125]. Consistent with these findings, we have previously reported that the lack of PS function increases axodendritic contacts, which was accompanied by increases of PSD-95 clusters, spine-like protrusions, and AMPA receptors-mediated synaptic transmission [71, 91]. Moreover, PS1 KO neurons and Wt neurons treated with *γ*-secretase inhibitors exhibited increases in the extent of cAMP/PKA activation [71, 91]. cAMP/PKA signaling plays a critical role in regulating short and long-term synaptic physiology [126]. It has been demonstrated that stimulus-induced activation of PKA pathway can also affect the synaptic morphology; therefore, it can indirectly affect basal synaptic transmission [127]. Thus, there exists a close relationship between increased phosphorylation of PKA substrates and enhanced synaptic transmission in neurons lacking PS function [71, 91]. 

**Figure 1:**
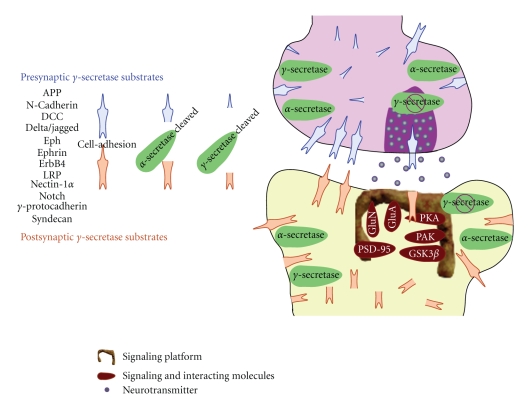
Schematic representation of PS-dependent processing of substrates and their role in synaptic function. Several *γ*-secretase substrates are located at the synapse where they influence the function of other synaptic proteins. Lack of *γ*-secretase-dependent cleavage of substrates could perturb presynaptic release and postsynaptic function of glutamate receptor-mediated events (NMDA-GluN and AMPA-GluA receptors). Synaptic contact could also be modulated through cell-adhesion properties of several *γ*-secretase substrates. Inefficient processing of these substrates will lead to sustained activation of signaling cascades capable of altering the postsynaptic morphology. How FAD-linked mutations in PS influence these processes and contribute to the disease progression has not been fully understood.

Signaling downstream of DCC, the netrin receptor [105], is also modulated by *γ*-secretase activity [71]. Upon binding of the ligand netrin, DCC undergoes metalloprotease-dependent ectocomain shedding [128], which generates a membrane-tethered DCC C-terminal fragment (CTF) derivative, consisting of the transmembrane segment and the intracellular domain. DCC CTF undergoes intramembraneous proteolysis by *γ*-secretase, and accumulation of DCC CTF in neuroblastoma cells treated with *γ*-secretase inhibitors stimulates neurite outgrowth [71, 129]. *γ*-secretase processing of DCC attenuates cAMP-dependent signaling cascades associated with DCC CTF [71]. In this case, it is clear that *γ*-secretase terminates intracellular signaling associated with DCC. However, it remains to be determined if *γ*-secretase cleavage of other substrates would significantly impact cellular functions, especially pertaining to synaptic process, through termination of receptor-mediated signaling events (see our proposed model in Figure 1).

More recently, it was found that EphA4 undergoes PS-dependent endoproteolytic process, and EphA4 CTF accumulates following inhibition of *γ*-secretase activity or in cells lacking PS expression [45]. Accumulation of EphA4 CTF was found tightly linked to an increase of spine-like protrusions in hippocampal cultures. Overexpression of an inactive Rac form abolished the enhancement of dendritic spines in neurons and lamellipodia formation in NIH3T3 cell lines. In addition, this study showed that overexpression of membrane-tethered EphA4 intracellular domain was also associated with an increase of lamellipodia formation in NIH3T3 cell lines. All together, these results suggest that enhanced accumulation of EphA4 intracellular domain may induce Rac-dependent signaling events that regulate cell morphology. 

It is clear that loss of intramembraneous proteolysis of *γ*-secretase substrates leads to the accumulation of their membrane-tethered cytosolic domains. The CTFs of certain substrates might serve as membrane anchors to facilitate the recruitment of signaling proteins in a manner that enhances phosphorylation of downstream signaling substrates. One of the signalings that have been implicated with PS function is GSK3*β* (reviewed in [130]). It is well established that PS1 can interact with the GSK3*β*/*β*-catenin complex [131–133]. However, besides this direct physical interaction with PS1, it is known that GSK3*β* is a ligand-receptor signaling molecule downstream of the activation of phosphatidylinositol-3-kinase pathway (reviewed in [134]). Specifically, it has been shown that GSK3*β* signaling is important for axon specification and elongation during the establishment of neuronal polarity (review by [135]). In addition, it has been reported that decrease of GSK3*β* activity parallels LTP induction paradigms, whereas inhibition of phosphatidylinositol-3-kinase and subsequent activation of GSK3*β* lead to decrease of LTP ([136]; reviewed in [130, 137]). Decreased phosphorylation of GSK3*β* at the Ser 9 residue, indicative of an increase of GSK activity, was also observed in PS1-deficient neurons as well as in PS1 neurons carrying FAD-linked mutations [69, 138–141]. Alteration of phosphatidylinositol-3-kinase /Akt signaling cascade has been proposed to be the link between GSK3*β* activity and PS function [138, 140, 142]. Interestingly, increase of GSK3*β* activity also leads to hyperphosphorylation of tau protein, which underlies one of the known pathological hallmarks of AD, namely the tangle formation (reviewed in [130]). 

It has been proposed that membrane microdomains rich in cholesterol and sphingolipids, termed lipid rafts, might influence *γ*-secretase activity and processing of substrates (reviewed in [143]). Lipid rafts play an important role in the maintenance of synapses through dendritic spine formation and AMPA receptor function [144]. Raft-dependent mechanisms facilitate trafficking of receptors in and out of the synapse and regulate synapse function (reviewed in [145]). Lipid rafts are known to serve as membrane platforms that compartmentalize diverse receptor-mediated signaling. Indeed, it was found that critical regulation of signaling associated with ErbB4, DCC, and EphA4, three *γ*-secretase substrates, involves their recruitment into lipid raft microdomains [45, 146, 147]. Based on the differences in spatiotemporal distribution of *γ*-secretase complexes and substrates [148, 149], different PS-dependent substrates might be subjected to different level of proteolysis depending on their membrane microdomain distribution at a given time during embryonic development and in adult life.

## 6. PS and Calcium Signaling

Besides a direct interaction of *γ*-secretase substrates with intracellular phosphorylation cascades, one of the key features of PS function is its role in intracellular Ca^2+^ homeostasis (reviewed in [22, 150, 151]). Ca^2+^ homeostasis is essential to maintain healthy cellular dynamics leading to proper physiological functions. Several studies have concluded that FAD-linked PS mutant expression in transfected cells and cultured neurons is associated with enhanced Ca^2+^ release from endoplasmic reticulum store. It has been reported that neurons generated from *PSEN1* M146V KI mice exhibit an increase of IP_3_-evoked Ca^2+^ responses in brain slices as early as in one month old [152]. This Ca^2+^ dysregulation appears to be specific to intracellular endoplasmic reticulum store since it does not affect the voltage-gated Ca^2+^ entry. However, it has been shown that L-type Ca^2+^ channel may be involved after stress induction at the neuromuscular junction in drosophila larvae expressing FAD-linked PS1 mutant [153]. Accordingly, in this model system, the level of synaptic plasticity and memory paradigm was normal following heat shock stimulation or endoplasmic reticulum stress, but reduced after 24 h of stimulation recovery. These results suggest that mutation in PS might alter synaptic behavior following recovery of stress conditions. It has been also proposed that PS might serve as a passive Ca^2+^ leak channel in the endoplasmic reticulum and FAD-linked PS variants might fail to exhibit this property [154]. Using reconstituted planar lipid bilayers, Tu and collaborators demonstrated that PS by itself could form low-conductance divalent ion channels, which was not the case in several mutated forms of PS. It remains to be determined if results from these experimental conditions are applicable to *in vivo* situations that are relevant to the disease state. 

More recently, Stutzmann and collaborators have established that the ryanodine receptor-evoked Ca^2+^ release (especially through RyR2 isoform) was increased in CA1 hippocampal slices of *PSEN1* M146V KI mice coexpressing Swedish APP and hyperphosphorylated tau mutants [155]. As a consequence, they observed an aberrant increase of ryanodine-dependent presynaptic neurotransmission, along with increases of long-term synaptic plasticity. Conversely, Shen and collaborators have observed a decrease of ryanodine-dependent presynaptic release in hippocampal neurons of PS-deficient mice [75]. All together, Stutzmann group concluded from their study that significant Ca^2+^ alterations are present at an early age even though Ca^2+^ homeostasis appears to be maintained. Compensatory mechanisms seem likely to take place in order to maintain normal synaptic function in early age. However, these subtle Ca^2+^-mediated alterations may have profound impact later on that can affect synaptic and cognitive functions in disease states.

## 7. Conclusions

Production and deposition of A*β* peptides clearly have central role in AD pathogenesis. However, it is becoming clear that FAD-linked mutations in PS proteins affect diverse physiological processes in addition to promoting the production of highly fibrillogenic A*β*42 peptides. The identification and characterization of *γ*-secretase substrates and the mechanistic details on the successive cleavage of substrates by the *γ*-secretase have enhanced our understanding of how partial loss-of-function associated with FAD-linked PS mutations can in fact lead to a gain of activities with reference to intracellular signaling associated with certain substrates such as DCC, ErbB4, and EphA4. At least in some cases, lack of *γ*-secretase processing leads to profound changes in synaptic structure and functions as a consequence of sustained intracellular signaling by substrate CTFs. As details begin to emerge on additional *γ*-secretase substrates, it will be possible to determine whether *γ*-secretase cleavage of neuronal receptors is indeed a regulatory step that modulates physiological signaling downstream of ligand binding and ectodomain shedding. Still, the major task is to establish whether or not altered signaling directly contributes to AD pathogenesis and/or AD-related synaptic dysfunction.
